# Safety in intrahospital transportation: evaluation of respiratory and hemodynamic parameters. A prospective cohort study

**DOI:** 10.1590/S1516-31802008000600005

**Published:** 2008-11-06

**Authors:** Bruno Franco Mazza, José Luiz Gomes do Amaral, Heloisa Rosseti, Rosana Borges Carvalho, Ana Paula Resque Senna, Hélio Penna Guimarães, Flavia Ribeiro Machado

**Keywords:** Patient transfer, Transportation of patients, Respiration, artificial, Positive-pressure respiration, Pulmonary ventilation, Intermittent positive-pressure ventilation, Transferência de pacientes, Transporte de pacientes, Respiração artificial, Respiração com pressão positiva, Ventilação pulmonar, Ventilação com pressão positiva intermitente

## Abstract

**CONTEXT AND OBJECTIVE:**

Intrahospital transportation of mechanically ventilated patients is a high-risk situation. We aimed to determine whether transfers could be safely performed by using a transportation routine.

**DESIGN AND SETTING:**

Prospective cohort study with "before and after" evaluation.

**METHODS:**

Mechanically ventilated patients who needed transportation were included. Hemodynamic and respiratory parameters were measured before and after transportation. Statistical analysis consisted of variance analysis and paired Student's t test. Results were considered significant if P ≤ 0.05.

**RESULTS:**

We studied 37 transfers of 26 patients (12 female) of mean age 46.6 ± 15.7. Patients with pulmonary diseases, positive end expiratory pressure > 5, FiO_2_ > 0.4 and vasoactive drug use comprised 42.4%, 24.3%, 21.6% and 33.0% of cases, respectively. Mean duration of transportation was 43.4 ± 18.9 minutes. Complications occurred in 32.4%. There was a significant increase in CO_2_ (before transportation, 29.6 ± 7.3 and after transportation, 34.9 ± 7.0; P = 0.000); a trend towards improved PO_2_/FiO_2_ ratio (before transportation, 318.0 ± 137.0 and after transportation, 356.8 ± 119.9; P = 0.053); increased heart rate (before transportation, 80.9 ± 18.7 and after transportation, 85.5 ± 17.6; P = 0.08); and no significant change in mean arterial blood pressure (P = 0.93).

**CONCLUSION:**

These results suggest that intrahospital transportation can be safely performed. Our low incidence of complications was possibly related to both the presence of a multidisciplinary transportation team and proper equipment.

## RESUMO

**CONTEXTO E OBJETIVO:**

O transporte intra-hospitalar de pacientes sob ventilação mecânica (VM) é uma situação sabidamente de alto risco. Nosso objetivo foi determinar se o transporte poderia ser realizado com segurança, seguindo uma rotina de transporte.

**TIPO DE ESTUDO E LOCAL:**

Estudo prospectivo, de coorte único com análise "antes e depois".

**MÉTODOS:**

Foram incluídos pacientes sob VM que necessitaram de transporte. Os parâmetros hemodinâmicos e respiratórios foram medidos antes (AT) e após (DT) o retorno à Unidade de Terapia Intensiva. Utilizou-se análise de variância e o teste t de Student. Os resultados foram considerados significativos se P ≤ 0,05.

**RESULTADOS:**

Foram avaliados 37 transportes em 26 pacientes (12 mulheres) com idade média de 46,6 ± 15,7. Pacientes com doença pulmonar, pressão expiratória final positiva > 5 cmH_2_O, FiO_2_ > 0,4 ou em uso de drogas vasoativas compreenderam 42,4%, 24,3%, 21,6% e 33,0% dos casos, respectivamente. A duração média do transporte foi de 43,4 ± 18,9 min. Complicações ocorreram em 32,4% dos casos. Houve aumento significativo no CO_2_ (AT - 29,59 ± 7,27 e DT - 34,95 ± 7,01, P = 0,000), tendência na melhora da relação PO_2_/FiO_2_ após o transporte (AT- 318,0 ± 137,0 e DT- 356,8 ± 119,9, P = 0,053) e aumento na freqüência cardíaca (AT- 80,9 ± 18,7 e DT- 85,5 ± 17.6, P = 0,08), sem alterações significativas na pressão arterial média (P = 0,93).

**CONCLUSÃO:**

Estes resultados sugerem que o transporte intra-hospitalar pode ser executado com segurança. Nossa baixa incidência das complicações é relacionada possivelmente à presença de uma equipe multidisciplinar de transporte e ao equipamento apropriado.

## INTRODUCTION

Over recent years, patients in intensive care units (ICUs) have become increasingly ill. One of the reasons for this is the high quality of life support that allows patients with multiple organ dysfunctions to survive. These patients usually present many comorbidities, including immunosuppression, organ transplantations, acquired immunodeficiency syndrome, chronic kidney or liver failure and neoplasia. The increasing severity of such illnesses has also been accompanied by a greater need for high precision in diagnoses. Moreover, the quality of support today allows even severely ill patients to undergo emergency surgery. They often undergo invasive procedures, inside and outside the ICU. This means that there is an increasing need to transfer severely ill patients out of the ICU, mostly for surgical or radiological procedures.^1^

Intrahospital transportation of ICU patients is a particular challenge, because of the severity of the illnesses and the need for continuous therapies during transportation, particularly mechanical ventilation. This has led to the development of specific monitoring and ventilatory equipment that is designed to manage this situation. This type of intrahospital transportation is associated with a high incidence of complications, which mostly relate to patient conditions or equipment problems.^2-6^

Many papers have been published regarding this subject. Some of them focused on gas exchange parameters, oxygen saturation and hemodynamic changes during transportation using a specific type of equipment,^7^ or assessed a specific subset of patients,^8-12^ such as neurosurgical patients.^13^ Others focused on the long-term consequences of transportation on oxygenation,^14^ and on the increased risk of ventilator-associated pneumonia.^15,16^

Nonetheless, one of the most noteworthy aspects of intrahospital transportation is the quality of the transportation team. It is reasonable to suppose that even dangerous conditions of this nature can be more easily handled if a multiprofessional team is available to manage any possible complication during the procedure. In 2004, guidelines for transfers of critically ill patients were reported. It has been suggested that a minimum of two people, one of them a critical care nurse, should accompany the patient. A medical doctor is certainly required for situations of caring for unstable patients.^17^

## OBJECTIVE

This study aimed to determine whether transportation could be safely performed, with regard to hemodynamic and respiratory parameters, by using a defined transportation routine that included the use of state-of-the-art ventilatory and monitoring equipment for transportation, as well as a multiprofessional team composed of a doctor, a nurse and a respiratory physiotherapist.

## METHODS

This was a prospective cohort study with "before and after" evaluation. All the patients were recruited from a 21-bed general ICU in a tertiary university hospital. Patients were included if they were over 18 years old, undergoing mechanical ventilation and needed to be transferred out of the ICU to the operating room, either for radiological investigation or for procedures with an expected duration of up to two hours. Patients who were in the anesthesiology recovery room and needed to be transferred to the ICU were also included provided they met further inclusion criteria. Patients who needed to be transferred outside of the hospital were excluded. The protocol was approved by the institution's ethics committee and all patients or their legal representative signed an informed consent form.

All transfers used ventilators equipped with microprocessors (Microtak 920 Plus, Takaoka^®^) that were adjusted so as to maintain the same mode of ventilation as used before transportation. The patients were monitored by means of an oximeter and noninvasive arterial blood pressure and electrocardiographic monitoring (M.3000, Morya^®^). The transportation team was composed of a physician, a nurse and a physiotherapist. Hemodynamic and respiratory parameters were measured immediately before disconnecting the patient from the basal ventilator and, after returning to the ICU, immediately before disconnection from the transfer ventilator. In order to evaluate safety, any complication during transportation was registered. Within this context, the main complications analyzed were hypoxemia (defined as arterial oxygen saturation below 90%), accidental disconnection and hypotension (defined as mean arterial pressure below 65 mmHg). These complications were selected because they were considered life-threatening. Agitation was also registered since, although non-threatening, it is the most common complication during transportation.

A sample size of 30 was calculated, based on analysis of the partial pressure of oxygen/fraction of inspired oxygen ratio (PO_2_/FiO_2_), with a β power of 80% and α of 0.05, in order to detect non-inferiority using a one-sided Student's t-test with a margin of equivalence of 0.750 and a true difference between the mean and the reference value of 0.000. The data were drawn from a single population with a standard deviation of 5.0. Statistical analysis was performed by using variance analysis and paired Student's t tests. The results were considered significant if P ≤ 0.05.

Our sample was composed of a total of 37 transfers performed on 26 patients (12 female and 14 male) with mean age of 46.6 ± 15.7 years. The main causes of ICU admission were trauma (42.4%) and elective neurosurgery (24.2%). Patients with pulmonary disease comprised 42.4% of all transfers. The characteristics of the population are presented in Table 1.

**Table 1:** Characteristics of all patients

Patient number	Gender	Age	Diagnosis at ICU admission	Respiratory failure
01*	male	23	polytrauma	pulmonary contusion
02	male	80	stroke	no
03	female	47	elective neurosurgery	no
04	male	60	subarachnoid hemorrhage	no
05†	female	71	carotid endarterectomy (PO)	no
06	male	46	mouth carcinoma (PO)	no
07	female	38	cerebral aneurysm (PO)	no
08	female	42	cerebral hemorrhage (PO)	no
09	female	60	mesenteric thrombosis (PO)	ARDS
10	female	61	subarachnoid hemorrhage	pneumonia
11‡	female	56	cerebral aneurysm (PO)	no
12	female	26	renal neoplasia (PO)	ARDS
13	female	32	intracranial tumor (PO)	no
14	male	21	polytrauma	no
15†	male	56	polytrauma	no
16†	male	40	polytrauma	pulmonary contusion
17	male	65	intracranial tumor (PO)	no
18†	male	43	cerebral hemorrhage (PO)	no
19	male	56	pneumonia	ARDS
20	male	48	polytrauma	pulmonary contusion
21	male	63	myocardium infarction	no
22	male	33	polytrauma	no
23	female	49	polytrauma	no
24	male	32	subarachnoid hemorrhage	no
25	female	40	preeclampsia	no
26	female	23	polytrauma	no

*ICU = intensive care unit; PO = postoperative (admission from the operating room); ARDS = acute respiratory distress syndrome. All patients had one transportation except as indicated:*

* *four*

† *two,*

‡ *three.*

## RESULTS

Most of the patients were transferred in order to perform a computed tomography (CT) scan (96.9%, mostly cranial). The mean duration of the transfer was 43.4 ± 18.9 minutes.

The ventilatory parameters before transportation (BT) and after transportation (AT) and the results from the blood gas analysis are shown in Table 2. Patients with pulmonary diseases, those ventilated with positive end-expiratory pressure (PEEP) > 5 and those with FiO_2_ > 0.4 comprised 42.4%, 24.3% and 21.6% of the cases, respectively. The mean PEEP and FiO_2_ levels before transportation were 6 and 0.4 respectively. A significant increase in CO_2_ was found: BT 29.6 ± 7.3; AT 34.9 ± 7.0 (P = 0.000). On the other hand, there was a trend towards an increased PO_2_/FiO_2_ ratio (BT 318.0 ± 137.0; AT 356.4 ± 119.9; P = 0.053).

**Table 2:** Ventilation parameters and blood gas analysis

Patient number	FiO_2_ (BT)	PEEP (BT)	PO_2_/FiO_2_ (BT)	PCO_2_ (BT)	PO_2_/FiO_2_ (AT)	PCO_2_ (AT)
01a	0.4	5	285	38	424	41
01b	0.4	10	190	36	322	45
01c	0.4	8	200	34	278	30
01d	0.5	10	282	27	460	29
02	0.5	5	238	28	224	29
03	0.4	5	415	27	306	33
04	0.3	5	276	33	328	39
05a	0.5	8	202	17	142	27
05b	0.5	5	246	25	254	31
05c	0.5	10	284	17	300	22
06	0.4	5	345	25	430	27
07	0.5	5	306	28	500	38
08	0.5	5	308	25	488	28
09a	0.4	10	235	28	308	41
10	0.4	5	405	35	476	41
11a	0.4	5	537	32	232	27
11b	0.4	5	340	19	392	27
12	0.4	8	272	46	354	47
13	0.4	5	400	44	558	49
14	0.4	5	537	26	666	42
15a	0.4	5	247	33	346	49
15b	0.4	5	255	33	222	36
15c	0.4	5	272	23	336	30
16a	0.4	5	177	34	334	36
16b	0.4	5	137	27	146	46
16c	0.4	5	170	36	298	42
17	0.4	5	407	29	466	31
18a	0.4	5	765	30	500	31
18b	0.4	5	277	32	422	38
19	0.4	10	235	32	224	34
20	0.5	12	220	40	200	38
21	0.4	5	547	21	364	31
22	0.4	5	147	29	194	29
23	0.4	5	400	26	444	28
24	0.3	5	583	12	492	33
25	0.4	5	312	30	408	32
26	0.4	5	432	38	362	36

*BT = before transportation; AT = after transportation.*

*FiO_2_ = fraction of inspired oxygen; PO_2_ = partial pressure of oxygen; PCO_2_ = partial pressure of carbon dioxide; PEEP = positive end expiratory pressure.*

In 33.0% of the cases, vasoactive drugs were used before and during transportation. There was a trend towards an increased heart rate (BT 80.9 ± 18.7; AT 85.5 ± 17.6; P = 0.08) with no significant changes in the mean arterial blood pressure (P = 0.93). Complications occurred in only 32.4% of all transfers and consisted mainly of agitation (66.7%), which was easily treated with an increase in sedation. Other complications were transitory and considered non-serious (Table 3).

**Table 3:** Complications during transportation

Complication	n	%
Agitation	8	66.7
Hypotension	2	16.7
Hypertension	1	8.3
Sat O_2_ < 90%	1	8.3

## DISCUSSION

This study was able to show that the transportation of critically ill patients can be safely performed, and without major changes in respiratory or hemodynamic parameters.

We found that our incidence of complications was very low in comparison with other results in the literature. Some studies showed a higher rate of complications,^18-20^ including transportation-related death.^19^ The very low incidence of complications that we found, even among the patients with respiratory failure, suggests that transfer out of the ICU should not be withheld due to the condition of these patients. It seems relatively safe to carry out procedures or examinations that could lead to better care for these patients. In a cohort of 103 consecutive transfers for diagnostic evaluations on trauma patients, the results from examinations or procedures led to a change of therapy in 24% of the cases.^21^ In another study, Hurst et al. showed changes in patient management in 39% of cases. The main reasons for the diagnostic procedure were follow up (37%), identification of a septic focus (34%) and identification of the bleeding site (14%); the most efficient examinations were angiography and abdominal CT, resulting in therapeutic consequences for more than 50% of the patients.^22^

Respiratory equipment appears to be one of the leading causes of complications during transportation. Worsening of oxygenation and ventilation, interrupted cycling and disconnections are the most threatening events. Therefore, it is reasonable to suppose that our low incidence of complications and the good blood gas parameters found in this study were partially due to the good performance of our transportation ventilator. Together with this, the monitoring equipment is important for ensuring a complete hemodynamic evaluation on the patient and for detecting any hypoxemia, hypotension or cardiac arrhythmias that might worsen the respiratory function.

Another important difference in our protocol was the use of a multidisciplinary team for all transfers. In our study, the transportation team for all transfers included a physician, a nurse and a respiratory therapist. Some authors have used incremental increases in the numbers of skilled personnel in the transportation team, depending on the severity of the illness.^6^ It is not common for all transfers to be accompanied by a physician, and this may have accounted for the low incidence of complications and the minimal variation in the oxygenation and ventilation parameters in our study. In another study that reported the personnel involved in transportation, at least two people accompanied the patient, but the physician was replaced with a respiratory therapist in 17.8 to 58% of transfers.^3^ These data suggest that the complications ought to be reduced when a transportation team has received specific training for managing critically ill patients.

As the ventilatory parameters remained unchanged during transportation, we expected the blood gas analysis not to show great differences. However, although without reaching a significant statistical difference, the oxygenation results after transportation appeared to be better than previously. This could be secondary to better sedation achieved during transportation or a slight difference in PEEP applied by the transportation ventilator.

The mean PEEP and FiO_2_ levels before transportation were 6 and 0.4 respectively, and the mean age was 46.6 ± 15.7 years. Marx et al. found that age greater than 43 and FiO_2_ > 0.5 were predictors of respiratory deterioration during transportation.^14^ The transportation itself must be as safe as possible, and it should not imply additional risks for the patient. Circulatory and ventilatory problems may arise during intrahospital transportation of critically ill patients, and therefore it is important for the transportation team to be properly trained for this kind of situation. In order to prevent adverse effects in this situation, the transportation organization, including the transportation team, equipment and monitoring systems, must be checked before making the transfer.

Our study had one important limitation: the small population, which may have limited our power to show differences in respiratory and hemodynamic parameters. However, the sample size was similar to those of other studies in the literature. Moreover, it was a well-designed prospective study.

## CONCLUSION

These results suggest that intrahospital transportation can be safely performed, with no hemodynamic and respiratory changes. Our low incidence of complications was possibly related to both the presence of a multidisciplinary transportation team and to proper equipment. In conclusion, it is important to optimize transportation, since this is a crucial problem among ICU patients who need to be frequently removed from the ICU for procedures that may enhance survival. While aiming to achieve this objective, we should not impose on our patients any further risk caused by a lack of high transportation standards. A multiprofessional team, together with high quality equipment, can help to reduce transportation-associated morbidity and mortality.

## References

[1] Stevenson VW, Haas CF, Wahl WL (2002). Intrahospital transport of the adult mechanically ventilated patient. Respir Care Clin N Am.

[2] Lovell MA, Mudaliar MY, Klineberg PL (2001). Intrahospital transport of critically ill patients: complications and difficulties. Anaesth Intensive Care.

[3] Waydhas C (1999). Intrahospital transport of critically ill patients. Crit Care.

[4] Fromm RE, Dellinger RP (1992). Transport of critically ill patients. J Intensive Care Med.

[5] Branson RD (1992). Intrahospital transport of critically ill, mechanically ventilated patients. Respir Care.

[6] Szem JW, Hydo LJ, Fischer E, Kapur S, Klemperer J, Barie PS (1995). High-risk intrahospital transport of critically ill patients: safety and outcome of the necessary "road trip". Crit Care Med.

[7] Evans A, Winslow EH (1995). Oxygen saturation and hemodynamic response in critically ill, mechanically ventilated adults during intrahospital transport. Am J Crit Care.

[8] Romano M, Raabe OG, Walby W, Albertson TE (2000). The stability of arterial blood gases during transportation of patients using the RespirTech PRO. Am J Emerg Med.

[9] Barton AC, Tuttle-Newhall JE, Szalados JE (1997). Portable power supply for continuous mechanical ventilation during intrahospital transport of critically ill patients with ARDS. Chest.

[10] Johannigman JA, Branson RD, Campbell R, Hurst JM (1990). Laboratory and clinical evaluation of the MAX transport ventilator. Respir Care.

[11] Miyoshi E, Fujino Y, Mashimo T, Nishimura M (2000). Performance of transport ventilator with patient-triggered ventilation. Chest.

[12] Nakamura T, Fujino Y, Uchiyama A, Mashimo T, Nishimura M (2003). Intrahospital transport of critically ill patients using ventilator with patient-triggering function. Chest.

[13] Bekar A, Ipekoglu Z, Türeyen K, Bilgin H, Korfali G, Korfali E (1998). Secondary insults during intrahospital transport of neurosurgical intensive care patients. Neurosurg Rev.

[14] Marx G, Vangerow B, Hecker H (1998). Predictors of respiratory function deterioration after transfer of critically ill patients. Intensive Care Med.

[15] Kollef MH, Von Harz B, Prentice D (1997). Patient transport from intensive care increases the risk of developing ventilator-associated pneumonia. Chest.

[16] Bercault N, Wolf M, Runge I, Fleury JC, Boulain T (2005). Intrahospital transport of critically ill ventilated patients: a risk factor for ventilator-associates pneumonia--a matched cohort study. Crit Care Med.

[17] Warren J, Fromm RE, Orr RA, Rotello LC, Horst HM, American College of Critical Care Medicine (2004). Guidelines for the inter- and intrahospital transport of critically ill patients. Crit Care Med.

[18] Braman SS, Dunn SM, Amico CA, Millman RP (1987). Complications of intrahospital transport in critically ill patients. Ann Intern Med.

[19] Waddell G (1975). Movement of criticall ill patients within hospital. Br Med J.

[20] Smith I, Fleming S, Cernaianu A (1990). Mishaps during transport from the intensive care unit. Crit Care Med.

[21] Indeck M, Peterson S, Smith J, Brotman S (1988). Risk, cost, and benefit of transporting ICU patients for special studies. J Trauma.

[22] Hurst JM, Davis K, Johnson DJ, Branson RD, Campbell RS, Branson PS (1992). Cost and complications during in-hospital transport of critically ill patients: a prospective cohort study. J Trauma.

